# Effect of formic acid treatment on colostrum quality, and on absorption and function of immunoglobulins: a randomized controlled trial in Holstein dairy calves

**DOI:** 10.1186/s12917-022-03418-x

**Published:** 2022-08-17

**Authors:** Billy I. Smith, Sarah V. Cady, Helen W. Aceto

**Affiliations:** Department of Clinical Studies – New Bolton Center, University of Pennsylvania, School of Veterinary Medicine, 382 West Street Road, Kennett Square, 19348 PA USA

**Keywords:** Randomized Controlled Trial, Acidified Colostrum, Pathogen Load, Neutralizing Capability

## Abstract

**Background:**

Good quality colostrum is characterized by high immunoglobulin concentration and low pathogen load. Some methods of pathogen reduction can decrease immunoglobulin concentration and potentially affect their function. Objectives were to determine the effect of formic acid treatment on colostral bacterial and immunoglobulin (IgG) levels before feeding, and serum immunoglobulin concentration and neutralizing capabilities after feeding. Fifteen female Holstein calf pairs born < 12 h apart from different dams were randomly assigned to receive four liters of either untreated pooled (both dams) colostrum (MC) or colostrum acidified to pH 4.0–4.5 (AC). Colostrum characteristics estimated; pH, bacterial load, IgG concentration, and neutralization of Infectious Bovine Rhinotracheitis (IBRV/BoHV-1), Bovine Viral Diarrhea (BVDV) Types 1 and 2. Blood samples were collected on days 1, 3 and monthly for 6 months and were analyzed for IgG, and both viral plus leptospiral neutralization, and total protein (day 3 only).

**Results:**

Compared to MC (mean 6.7, SD 0.4; median 6.8, range 6.0–7.3), AC pH was significantly reduced (mean 4.3, SD 0.2; median 4.3, range 4.0–4.5; *P* < 0.001). Total coliform count (cfu/mL) was also reduced (MC mean 149, SD 444; median 1, range 0–1,700; AC mean 8, SD 31; median 0, range 0–120; *P* = 0.02). Colostrum IgG concentration was not significantly different between MC (mean 93.3, SD 39.7; median 92.8, range 36.7–164.4 g/L) and AC (mean 101.9, SD 36.7; median 108.3, range 33.8–164.4 g/L; *P* = 0.54). In calves, serum IgG peaked on day 3 (MC mean 26.1, SD 34.9; median 169.2, range 8.3–151.0 g/L; AC mean 30.2, SD 48.7; median 188.8, range 3.1–204.4 g/L; *P* = 0.77), and apparent efficiency of IgG absorption was not different between groups (MC mean 24.3, SD 11.4, median 25.3, range 8.6–51.3%; AC mean 22.6, SD 21.7, median 21.6, range 4.1–58.9%; *P* = 0.65). Thereafter, IgG levels declined but did not differ between groups. MC and AC serum neutralizing titers for IBRV, BVDV Types 1 and 2, or *Leptospira interrogans* serovars Canicola, and Pomona and *L. borgpetersenii* serovar Hardjo were not different.

**Conclusions:**

Colostrum acidification significantly decreased bacterial load fed to newborn calves without affecting colostral IgG concentration or virus neutralization. In addition, acid treatment did not affect serum IgG concentration in calves or its activity against common pathogens.

## Background

According to the United States Department of Agriculture’s National Animal Health Monitoring System (NAHMS) 2014 Dairy Survey, gastrointestinal and respiratory problems are responsible for 56.4% and 24% of pre-weaned heifer deaths, respectively [1]. Additionally, respiratory disease is the predominant cause of mortality in weaned dairy heifers [1]. Calves are born agammaglobulinemic and their capacity to mount an immune response de novo is limited. Therefore adequate colostrum intake that leads to a successful transfer of passive immunity is critical for disease prevention and their survival [2–5]. The importance of absorbing an adequate quantity of immunoglobulins is unequivocal, but their functionality is also crucial to the health and welfare of dairy calves.

The cost of preventing and treating dairy calf disease contributes to economic losses, but the impact of calfhood disease on future productivity should also be considered. For example, a single case of pneumonia during the first 2 months of life results in slower growth rates due to poor animal health and welfare, and decreased productivity as an adult [6, 7]; improved rate of average daily gain has been associated with a higher first lactation milk yield [8].

Colostrum provides a favorable environment for bacterial growth. Contamination has a negative impact on the growth of dairy heifers and increases the risk of pre-weaning morbidity and mortality [9]. Moreover, it is known that increased pathogen load in colostrum interferes with immunoglobulin absorption leading to poor passive transfer [10–12]. Limiting exposure of calves to colostrum contaminated with pathogenic organisms; bacteria, including *Escherichia coli* [13], *Salmonella* spp. [14], *Mycoplasma bovis* [15, 16], *Listeria monocytogenes* [17], and *Mycobacterium avium* ssp. *paratuberculosis* [18], and viruses (Bovine Leukemia Virus [19]), is a key factor in disease prevention.

Pathogen reduction using techniques such as heat treatment, pasteurization, irradiation (ultraviolet light), can be complicated and costly, and may result in an unacceptable product because of increased viscosity and feed refusal. A critical negative effect that might also occur with these techniques is denaturing of immunoglobulins leading to impairment of passive transfer [20–22]. The magnitude of IgG loss following heat treatment may be dependent on the initial IgG concentration, with losses being greatest when concentrations are high [15]. Additionally, any structural changes likely limit pathogen neutralizing capabilities of those maternal antibodies that are transferred to the blood, thereby directly compromising both the quantity and quality of immunoglobulins administered to calves. For instance, heat treatment has been shown to damage bovine immunoglobulins by changing their secondary structure causing them to lose immunoactivity [23–25]. Even widely adopted heat-treatment techniques such as 60 min at 60 °C have been shown to decrease the abundance of immunoglobulins and other important components of colostrum likely to play a role in gut maturation, glucose absorption and mucosal immunity [26].

As an alternative to standard techniques, use of acids to acidify whole milk or milk replacers has recently gained attention in the dairy industry [27–33] Acidification might also be a means to control the growth of bacteria in colostrum. Even though acid use has been anecdotally successful and its popularity is increasing there are potential consequences. While the effect of acidified colostrum on IgG absorption has been reported in abstract form [34], we are not aware of any formal publications in the published literature describing possible functional effects on immunoglobulins. The aim of this study was to test the hypothesis that formic acid acidification could successfully reduce bacterial (pathogen) load without negatively impacting either the absorption or immunoactivity of colostral antibodies; thereby indicating that it could be an effective tool in colostrum management.

## Results

### Calf characteristics

The power analysis indicated that 10 calves per group would be sufficient to detect a minimal difference in titers between the two groups of 1:291 with alpha = 0.05 and power = 1.00. As 15 calves per group were used, the power of the study should have been sufficient to detect meaningful differences associated with feeding AC. A total of 15 calves were fed AC and 15 calves were fed MC, data collected from all 30 calves were included in the analysis. The mean birth weights for the 15 MC and 15 AC calves were not significantly different (*P* = 0.95, Table 1).

**Table 1:** Calf and colostrum characteristics for groups receiving 4 L of untreated (MC) or acidified (AC) colostrum

Parameter	Untreated Colostrum (*n* = 15)	Acidified Colostrum (*n* = 15)
Birth Weight (kg)	40.6 (2.4)	40.7 (2.9)
Colostrometer (g/L)	103 (21)	103 (21)
Colostrum Brix (%)	24.8 (3.1)	24.6 (3.2)
Colostrum pH (post-processing)^**a**^	6.8 (6.5–7.0)	4.3 (4.3–4.5)*
Colostrum IgG (g/L)	93.3 (39.7)	101.9 (36.7)
Serum Total Protein (day 3, mg/dL)	6.5 (0.4)	6.3 (0.7)
Apparent Efficiency of IgG Absorption (AEA day 3, %)^**a**^	24.3 (14.7–30.0)	22.6 (13.6–28.9)

^**a**^All values are shown as mean and standard deviation (SD) except for colostrum pH and AEA which were not normally distributed and are shown as median and interquartile range (IQR, 25–75%)

^*^pH of acidified colostrum was significantly lower than untreated colostrum *P* < 0.001, Wilcoxon rank sum

### Colostrum characteristics

#### pH reduction

Compared to MC, the pH of AC was significantly reduced (*P* < 0.001, Table 1).

#### IgG concentration

The concentrations of IgG were not significantly different from MC after acidification (*P* = 0.54; Table 1). Both colostrometer (correlation coefficient *r* = 0.63, *P* < 0.001) and Brix refractometer (*r* = 0.64, *P* < 0.001) readings were substantively (moderately) and statistically significantly correlated with IgG concentrations measured by turbidometric immunoassay (TIA). Using a cutoff of 50 g/L of IgG measured by TIA as an acceptable standard for colostrum, the equivalent cutoffs were 86.8 g/L and 22.2% for the colostrometer and Brix refractometer, respectively.

#### IgG virus neutralizing titers

There were no significant differences between untreated and post-acidified colostrum in immunoglobulin levels against IBRV, BVDV Type-1, or BVDV Type-2 (Table 2).

**Table 2:** Colostrum and serum viral neutralizing titers over time for IBRV and BVD

Source	Day	IBRV Titer Median (IQR^a^)		BVDV Type-1 Titer Median (IQR^a^)		BVDV Type-2 Titer Median (IQR^a^)	
		Untreated (*n* = 15)	Acidified (*n* = 15)	Untreated (*n* = 15)	Acidified (*n* = 15)	Untreated (*n* = 15)	Acidified (*n* = 15)
Colostrum	1	256 (64–1024)	256 (128–512)	8192 (4096–8192)	8192 (8192–8192)	8192 (4096–8192)	8192 (8192–8192)
Blood (serum)	1	1 (1–1)	1 (1–1)	1 (1–1)	1 (1–1)	1 (1–1)	1 (1–1)
	3	32 (16–128)	32 (16–128)	1024 (512–2048)	1024 (512–4096)	2048 (1024–2048)	4096 (1024–4096)
	30	8 (8–32)	16 (8–32)	256 (128–256)	512 (256–1024)	512 (64–512)	1024 (512–2048)
	60	8 (4–16)	8 (4–16)	64 (64–128)	128 (64–256)	128 (64–512)	256 (64–1024)
	90	2 (2–4)	4 (1–4)	32 (8–32)	32 (16–64)	32 (16–64)	64 (16–128)
	120	1 (1–2)	1 (1–2)	4 (2–8)	8 (4–16)	8 (4–16)	16 (8–64)
	150	1 (1–1)	1 (1–1)	2 (1–2)	2 (2–4)	4 (1–8)	4 (1–16)
	180	1 (1–1)	1 (1–1)	1 (1–2)	1 (1–1)	1 (1–2)	2 (1–4)

^a^*IQR* Interquartile Range 25–75%

#### Microbial reduction

**Fig. 1:**
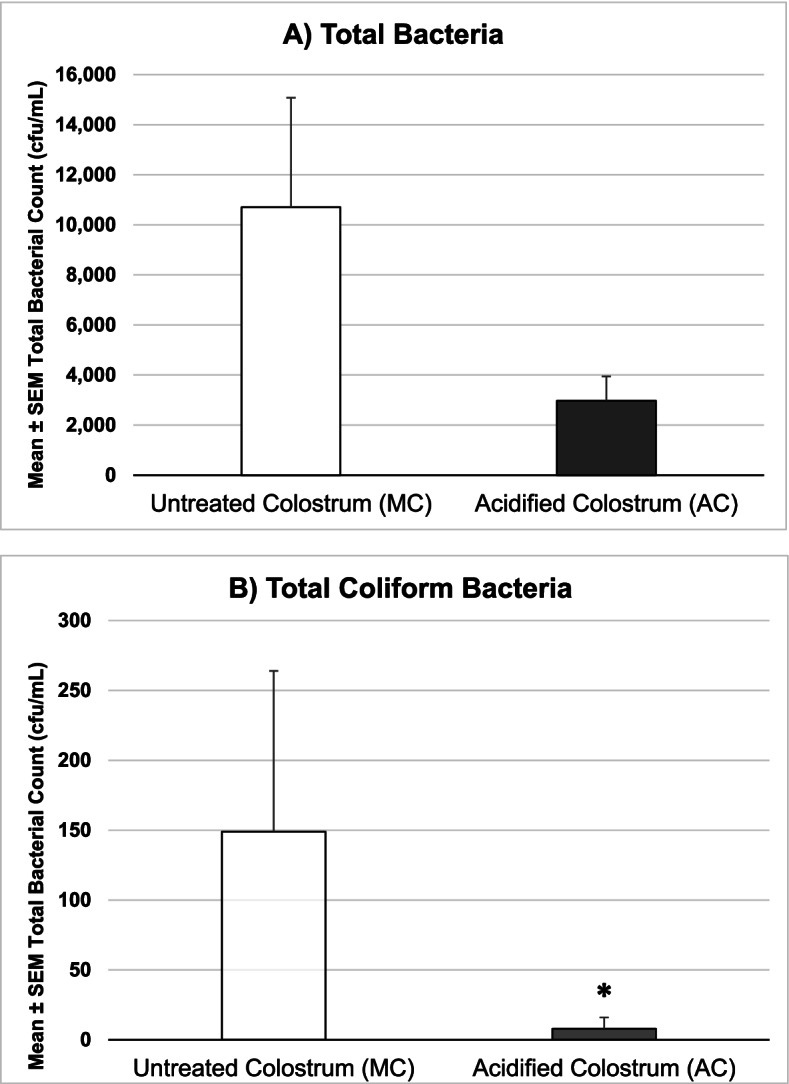
Effect of acidification of colostrum on **A**) total and **B**) coliform bacterial counts (*n* = 15 per group). Data are plotted as mean ± standard error but were analyzed using non-parametric methods which make no assumptions about data distributions. * Total coliform count was significantly reduced in acidified compared to untreated colostrum *P* = 0.02, total bacterial count was also reduced but the effect was not statistically significant; Wilcoxon Rank Sum Test

Compared to MC, total bacterial counts in AC were reduced (MC mean 10,700, SD 16,945; median 3,700, range 1,000–62,300; AC 2,973, SD 3,765; range 1,000–12,200) but this effect did not achieve statistical significance *P* = 0.10 (Fig. 1). There was, however, a significant reduction in the total coliform count (MC mean 149, SD 444; range 0–1,700; AC mean 8, SD 31, range 0–120; *P* = 0.02; Fig. 1). Generic *E. coli* were rarely detected and there was no significant difference between groups (MC mean 6, SD 18, range 0–70, AC not detected; *P* = 0.48).

### Serum characteristics

#### Total protein concentration

At 3 days of age, total protein levels in calves fed MC and those receiving AC were not significantly different (*P* = 0.38; Table 1). On day 3, the total serum protein concentration (refractometer) and serum IgG concentration (TIA) were significantly correlated both substantively (*r* = 0.67) and statistically (*P* < 0.001). Cutoffs for serum IgG concentrations measured by TIA equivalent 5 g/dL and 5.5 g/dL of serum total protein, well recognized as being indicative of good and excellent passive transfer, were 8.1 g/L and 11.6 g/L, respectively. Of the 30 study calves none had a total protein < 5 g/L and only 3 were between 5 and 5.5 (2 MC, 1 AC), one value was missing. IgG concentrations on day 3 were > 11.6 g/L in 26/30 calves, and between 8.1 and 11.6 g/L in 3 calves (2 MC, 1 AC). Only one AC calf at 3.1 g/L had poor passive transfer according to the TIA concentration cutoff calculated from the regression and there was a mismatch with its total protein of 5.2 g/dL.

#### IgG concentration

**Fig. 2:**
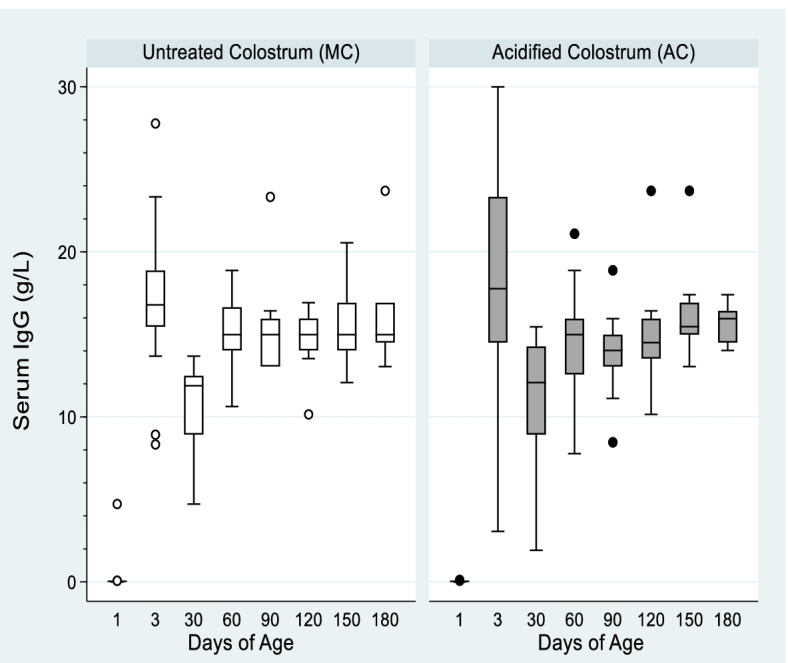
Box and whisker plot of median serum immunoglobulin (IgG) levels over time measured by turbidometric immunoassay (TIA). Calves were fed 4 L of untreated (MC; *n* = 15) or formic acid acidified (AC; *n* = 15) colostrum at birth; the blood sample on day 1 was collected immediately prior to colostrum feeding

With the exception of one calf in the MC group (4.7 g/dL), IgG levels on day 1 pre-colostrum were all less than 0.1 g/L. Peak IgG occurred on day 3 i.e., approximately 72 h after feeding (MC mean 26.1, SD 34.9, range 8.3–151.0 g/L; AC mean 30.2 SD 48.7, range 3.1–204.4 g/L; *P* = 0.77; day 3 regression coefficient 0.9, 95% CI -3.6–5.5; *P* = 0.67; Fig. 2). Thereafter IgG levels slowly declined but they were never significantly different between groups at any time point. Regression analysis of the entire time course post-colostrum (i.e., days 3–180) also failed to demonstrate a significant difference between groups (coefficient 0.0005, 95% confidence interval [CI] -0.022–0.023 g/L, *P* = 0.97; Fig. 2). On the most critical day i.e., day 3, apparent efficiency of IgG absorption (AEA) was not different between MC and AC groups (*P* = 0.65; Table 1: day 3, coefficient -1.3, 95% CI -8.6–5.9, *P* = 0.71).

#### IgG serum neutralizing titers

There were no significant differences between MC and AC in immunoglobulin SNTs for IBRV, BVDV Type-1, or BVDV Type-2 at any point over the 180-day observation period (Table 2). Maternal antibody titers against IBRV, BVDV Types 1 and 2 tended to remain higher for longer in calves that received AC compared to calves that were fed MC, but this effect did not achieve statistical significance even when tobit regression was used to examine the overall time course (IBRV *P* = 0.53; BVDV Type-1 *P* = 0. 35; BVDV Type-2 *P* = 0.20: Table 2).

SNTs against *Leptospira interrogans* serovars Grippotyphosa and Icterohaemorrhagiae were rarely observed and were not subject to analysis. *L. interrogans* serovars Canicola and Pomona SNTs were infrequently detected in both groups and at no time were significantly different between those calves fed MC or AC (tobit regression over time course *P* = 1.0). SNT’s against *L. borgpetersenii* serovar Hardjo were measurable in all except one AC calf at day 3 and were still detectable in 37% of the calves at day 60, after which only one or two calves had measurable titers. There was no significant difference between MC or AC groups at any time point nor over the entire observation period (tobit regression days 3–180, *P* = 0.39).

## Discussion

High bacterial counts in colostrum can impact calf health both directly and by negatively impacting immunoglobulin absorption [11, 12, 35]. Fresh/raw colostrum fed to calves should contain less than 100,000 cfu/mL total bacterial count and less than 10,000 cfu/mL total coliform count [4, 5]. However, these levels are often exceeded, for example, an observational study in which 827 colostrum samples from 67 farms in 12 states were tested, reported almost 43% of samples had total plate counts greater than 100,000 cfu/mL and 17% of samples had greater than 1 million cfu/mL [36]. Strategies to reduce bacterial counts in colostrum are indicated. The current study lends support to the use of acidification of bovine colostrum to improve colostrum quality through bacterial reduction. Acid is already being used to manipulate whole milk or milk replacer for calves [27–33] and was chosen here partly because of its increasing popularity. Moreover, it is a relatively simple and inexpensive technique accessible to most producers. Acidification has already been shown to reduce bacterial load including some specific pathogens [34, 37–39]. However, it has some important drawbacks including danger of handling, palatability of end-product (viscosity and taste), and potential damage to colostrum components, specifically immunoglobulins. Nevertheless, acidification offers an additional advantage to some other methods of colostrum treatment in that it may permit prolonged storage without the need for freezing or refrigeration. However, extended shelf-life would only be of value if it could be demonstrated that acid decreased bacterial load, without negatively affecting levels, absorption, and neutralizing capability of colostral immunoglobulins. Research into the effects of feeding acidified colostrum to newborn Holstein dairy calves is limited, particularly in terms of functional outcomes. This study showed that acidification of colostrum reduced bacterial load, potentially including pathogens (total coliforms), without negatively affecting immunoglobulins or their ability to neutralize selected pathogens after feeding to newborn calves.

Handling of acid should be done with care and eye and skin protective measures should always be used. United States Food and Drug Administration (FDA) regulations state that formic acid and formate salts from all added sources (if multiple sources of formic acid and its salts are used in combination) cannot exceed 1.2% of complete feed [40]. Although there are currently no guidelines for whole milk or colostrum, formic acid levels in all AC samples in this trial were ≤ 0.45%, i.e., approximately one third or lower than the established guidelines for livestock feeds. Throughout the study’s 6-month sample collection period, no ill-effects were noted in any calves that received AC and all study animals went on to be productive members of the participating herd. Moreover, this herd has years of experience feeding ad libitum acidified milk to calves and has never recorded any detrimental effects, including no impacts on intake such as those described in some studies (see below). Observations made during the current trial indicated that three factors chiefly contributed to successful colostrum acidification. First of all, it was important to chill the colostrum prior to addition of acid. Adding acid to colostrum with a temperature at or above 15–16 °C (60 °F) resulted in coagulation and increased viscosity, changes which make calves refuse to drink and essentially preclude esophageal tube feeding. Secondly, expect to use slightly more acid when acidifying colostrum compared to acidification of milk. For example, compared to the same volume of whole raw milk, we used 15 mL more (40–45 vs 25–30 mL) of the 10% acid solution per 4 L of colostrum to reach the desired pH (4.0 to 4.5). Finally, the colostrum must be vigorously stirred while slowly adding the acid solution. The use of a drill attachment designed for mixing paint worked well to rapidly mix the acid and colostrum thereby preventing coagulation.

The method we used, resulted in colostrum with a low bacterial concentration, that was stable at room temperature for days (unpublished observations and a currently funded research project), and acceptable to calves via bottle or esophageal tube feeding. The main drawback found with formic acid acidified colostrum was the effect on palatability, which is likely encountered with all acids. We have observed calves to occasionally reject suckling or take longer to consume the desired amount of acidified colostrum or milk when bottle-fed. Moreover, acidification with formic acid to pH levels used in the current study i.e., between 4.0 and 4.5, has been reported to limit voluntary intake of milk replacer by approximately 1 L/d [31]. Other studies have shown that some calves permitted to feed ad libitum, reject colostrum [28] or milk replacer acidified to pH below 4.5 [29] or exhibit fragmented feeding patterns [32]. In common with our own observations, these findings indicate that low pH can alter the palatability of milk or colostrum fed to calves. However, at least in the case of colostrum, that decrease in palatability and risk of rejection can be overcome by esophageal tube feeding which is not hindered in any way. In the current study, to ensure complete intake of 4 L, colostrum (MC and AC) was administered to all calves by esophageal feeder. Although acidification of milk replacer has been fairly widely used in feeding of dairy calves [27–33] there is a paucity of information on what impact if any acidification might have on the gastrointestinal microbiome of young calves. There is evidence that calves fed acidified milk replacer ad libitum have lower abomasal and fecal pH than calves fed restricted amounts of nonacidified milk replacer [41], raising the concern that lower gastric pH might negatively impact digestive function, or cause gastrointestinal discomfort. However, a study designed to evaluate feeding and other behaviors potentially indicative of GI discomfort with free-access feeding of acidified milk, found that apart from a possible effect on palatability, despite which there was no feed avoidance in the first week of life, no behavioral impacts were observed [32]. Interestingly, a recent study at our institution has shown that feeding calves acidified colostrum increased abundance of *Faecalibacterium* in the first week of life (Hennessy et al. unpublished data, June 2022), and *Faecalibacterium* is associated with decreased diarrhea and better calf growth [42, 43], suggesting that acidified colostrum might actually enhance GI function. To be considered excellent quality, colostrum should have a low bacterial (including pathogens) load, but the process of harvesting colostrum could result in bacterial contamination. Furthermore, cattle suffering from mammary gland infections at the time of calving could also contribute additional bacteria to the collected colostrum. In addition to bacterial pathogens, colostrum can also be a channel of transfer for certain viruses [44]. The results of this study showed what might well be a clinically meaningful reduction in total bacteria and more specifically a significant reduction in total coliforms. Reduction of pathogens is a critical component of colostrum management because they are known to bind to free immunoglobulin in the gut lumen or block immunoglobulin uptake and transport across intestinal epithelial cells, thereby interfering with absorption [5, 10]. Decreasing bacteria to remove potential pathogens is permissive to enhanced passive transfer [5, 10, 12], and subsequent promotion of calf health. From a microbial standpoint, the colostrum on our well-managed study farm was of excellent quality from the outset, with ranges for total bacterial and total coliform counts (1,000–62,300 and 0–1,700 cfu/mL, respectively) being well below published targets for untreated colostrum. Nonetheless, levels of bacteria and specifically coliforms were, reduced by acidification suggesting that routine use of acid might be a valuable tool in bacterial management. However, work with more heavily contaminated colostrum is necessary to definitively demonstrate this.

Estimation of the effect of acidification on immunoglobulin levels and on their immunoactivity both in colostrum and in serum after absorbtion were essential parts of this study. For measurement of IgG concentrations, we chose to use commercially available turbidometric immunoassay (TIA) kits. Available for bovine colostrum and bovine serum,^1^ advantages of which include accessibility, portability, simplicity, and cost-effectiveness. Although designed for point-of-care use, in this study all IgG concentrations were quantitatively determined spectrophotometrically in our clinical laboratory. Compared to the reference method of radial immunodiffusion (RID) and a point-of-care IgG ELISA, performance of the same manufacturer’s equine TIA test kit for measurement of immunoglobulins was good in quantifying equine serum IgG [45]. It might therefore be reasonable to expect that this methodology would perform just as well for measurement of bovine IgG. However, studies on comparative performance of RID and ELISA [46], ELISA and TIA [47], RID and Brix [48], RID, TIA and Brix [49], RID, Brix and colostrometer [50], RID and refractometer [51] have been somewhat inconsistent, with some studies showing relatively good comparative results [48, 49] while others report poor or contradictory correlations between tests and/or medium i.e., colostrum vs serum [46, 47, 50, 51] For example, Quigley et al. reported correlations between Brix and both TIA and RID, but TIA and RID, while correlated, were not consistent throughout the full concentration range of samples tested [49]. By contrast, Gelsinger et al. reported weak correlations between ELISA and RID results in plasma and unheated colostrum, such that IgG concentrations were significantly lower in all sample types when measured by ELISA, consequently they did not recommend direct comparison of ELISA and RID [46]. Although Schneider et al. found that colostrum IgG concentrations measured using the same test kit as that employed in the current study to be correlated with ELISA, the TIA values were on average 66.4% lower than those measured by ELISA and did not recommend use of TIA [47]. However, given the inconsistency of results across studies it is difficult to be definitive about the accuracy of the assays. When we compared correlations between colostrometer, Brix refractometer and TIA for IgG colostral concentrations, both colostrometer and Brix values were substantively and statistically correlated with TIA.

Other studies report Brix refractometry to be among the most consistent of methods available for measurement of colostral IgG [48, 49, 52]. Our data indicated that a Brix reading of 22.2% was equivalent to a colostral IgG concentration of 50 g/L measured by TIA, a value which is very similar to that reported for other studies [48, 49, 52]. By contrast, despite the apparent correlation, the colostrometer cutoff of 86.8 g/L did not closely match the TIA measurement. Moreover, examination of the scatter plots (not shown) revealed that for colostrometer readings only 5 samples were within 5 g/L of the fitted line for TIA IgG concentrations and 16 samples differed from the TIA measurement by ≥ 15 g/L. By contrast, 15 of the Brix readings were within 1% of the TIA fitted line and only 8 differed by more than 2% (i.e., approximately 5 g/L based on the 50 g/L cutoff being 22.2% Brix). In hindsight, it would have been better to have included Brix refractometry for assessment of serum IgG concentration, but we nevertheless feel that our use of TIA for both colostrum and serum is reasonable. In serum, total protein measured by refractometry and TIA were correlated and the total protein cutoffs of 5 and 5.5 g/dL often considered as gold standards for good and excellent passive transfer, respectively [4, 5, 53], generally matched the calculated equivalents for serum IgG concentration (8.1 and 11.6 g/L) measured by TIA. There was only one mismatch where an AC calf’s total protein of 5.2 g/dL did not match the serum IgG concentration of 3.1 g/L. Serum IgG measurements on subsequent days were also low indicating that the low level in this calf was a real effect. It is unclear why the total protein on day 3 was higher than might be expected based on the IgG concentration but could be related to the subjective nature of refractometry.

Colostrum quality has for the most part been judged by immunoglobulin concentration and bacterial load. The use of standard pathogen reducing techniques (heat treatment or true pasteurization, ultraviolet light irradiation, and pressure) are, under certain conditions, known to induce denaturing of immunoglobulins leading to reduced passive transfer and compromised serum immunoglobulin levels in calves [22–25]. Even maternal antibodies that do reach the blood of calves may have decreased immunoactivity. The ability of absorbed, undamaged immunoglobulin to neutralize pathogens is a key component in disease prevention leading to healthier calves. It has been reported that free-access feeding of acidified milk replacer results in improved growth and overall health of both dairy and veal calves [31]. Here we show that formic acid acidification of colostrum decreased bacterial load and was neutral in terms of its effect on immunoglobulin absorption measured as serum total protein and IgG concentrations, and AEA. More importantly, the neutralizing capabilities of the absorbed immunoglobulins against both viruses (IBRV, and BVDV Types 1 and 2) and bacteria (serovars of *Leptospira* spp) were not different between MC and AC groups suggesting that the immunoglobulins remained functionally intact after acidification. For IBRV the target titer for protection against the virus is ≥ 1:8 [54, 55], whereas those for BVDV types 1 and 2 are ≥ 1:128 [55, 56]. On day 3 all calves in this study had titers that exceeded the target levels for protection against said viruses.

Information gained from this project could benefit the production animal through supplementing existing management strategies directed at maintaining animal health and well-being. Additional studies to investigate the combined effect of feeding acidified colostrum followed by the provision of acidified whole milk or milk replacer on health in dairy youngstock and their subsequent productivity could further elucidate the benefits of acidification to the dairy industry. Future work is planned to investigate another potential benefit of acidification in the preservation of colostrum with reduced bacterial load without the need for freezing or refrigeration.

## Conclusions

Our results showed that acidification of colostrum decreased the bacterial load fed to newborn calves, including a significant reduction in total coliforms, which should aid in disease prevention. Acidification had no impact on absorption of immunoglobulins from the colostrum and neither did it negatively affect the neutralizing capability of maternal antibodies against common viral and bacterial pathogens.

## Methods

### Animal selection and colostrum collection

This study was conducted at a 520 milking Holstein dairy located in southeastern Pennsylvania, USA that routinely fed 4 L of raw pooled colostrum. The study herd was selected based on relatively large size, excellent computerized records (DairyComp 305, Dairy One Cooperative Inc., Ithaca, NY, USA), well-trained personnel (including one author, SVC, who is a veterinarian employed full-time by the farm and was responsible for the day-to-day conduct of the field trial), and willingness of the owner to comply with study requirements. Cows were moved to the maternity area, an open straw bedded pack, within one week of their due date. Records and careful monitoring were used to identify those cows likely to calve within 12 h of one another and from among these animals fifteen pairs of female Holstein calves were enrolled in the study from May–August 2018. In this high producing herd (rolling herd average 14,800 kg per cow annually; 4.1% fat and 3.2% protein), almost all cows routinely produce at least 8 L of colostrum. However, if the total volume of colostrum pooled from both dams was < 10 L the pair and their calves were not enrolled in the trial. Colostrum was harvested from both dams within 4 h of calving, collection was made over ice to rapidly cool to 15—20 °C. Once cooled, the colostrum from both dams was pooled. Four liters of the pooled colostrum was acidified as described below. Colostrum characteristics including pH, bacterial load, and immunoglobulin G (IgG) concentration were estimated in both untreated and acidified samples. Calves were removed from the dam immediately after birth and processed by navel dip, ear tagging, recording of body weight, and initial pre-colostrum blood draw. After processing, calf pairs were placed in a holding pen until being randomly assigned by coin-flip to receive via esophageal tube feeding 4 L of either untreated pooled maternal colostrum (MC) or the same pooled colostrum acidified to pH 4.0–4.5 with 10% formic acid (AC). Colostrum was not stored prior to feeding as all calf pairs received colostrum pooled and processed from their dams within 12 h of birth. After colostrum feeding, calves were housed in small groups (maximum 6); fed acidified whole milk twice daily and provided with water and a grain starter ration ad libitum until weaning at approximately 8 weeks of age. Post-weaning calves were housed in larger groups (maximum 15) and fed a grower ration before being transitioned onto a corn silage-based heifer mixed ration. In addition to day 1 pre-colostrum, blood samples were collected from each heifer pair on day 3 post-colostrum, and then monthly for 6 months. At no time during the study period did the enrolled calves receive any vaccinations. Blood was analyzed for IgG concentration and neutralizing capabilities against viral Infectious Bovine Rhinotracheitis (IBRV), Bovine Viral Diarrhea Virus (BVDV) Type-1 and Type-2 and five serovars of bacterial *Leptospira*. For analysis of samples, BIS and the participating laboratories were not aware of how the samples were coded.

### Colostrum acidification

Formic acid is available as an FDA approved food additive for livestock feed and drinking water (Code of Federal Regulations Title 21, 21CFR573.480, 2018) in clear liquid concentrated form and must be diluted to 10% with water before use as an acidifier. A 4 L aliquot of the cool pooled colostrum was acidified by slowly adding 10% formic acid while stirring vigorously until a pH between 4.0 to 4.5 was achieved. The volume of 10% formic acid required to reach the desired pH range was slightly variable but was between 40–45 ml/L and final concentration of formic acid in AC was ≤ 0.45%.

### Colostrum analysis

MC and AC were measured for immunoglobulin content by a colostrometer (Biogenics; Florence, OR) and a Brix refractometer (Cole-Parmer; Vernon Hill, IL) at the time of feeding i.e., immediately after pooling and processing of colostrum but within 12 h of the first calf in the pair being born. Measurement of pH also occurred at this time by means of Hydrion™ plastic indicator strips dipped into samples and read according to manufacturer’s instructions (Micro Essential Laboratory Inc.; Brooklyn, NY).

The concentration of IgG in refrigerated MC and AC was quantitatively determined spectrophotometrically within 48 h of initial processing using a commercially available turbidimetric immunoassay (TIA) validated for bovine colostrum IgG (DVM Rapid Test™ II, Value Diagnostics™, MAI Animal Health; Elmwood, WI).

Microbiological analysis of colostrum included counts (cfu/mL) for total bacteria, total coliforms, and generic *Escherichia coli*. A 30 ml aliquot of frozen MC and AC were shipped to the collaborating laboratory where they were thawed and inoculated onto aerobic and *E. coli* Petrifilm™ as described by the manufacturer (3 M US; Minneapolis, MN). Colostrum was diluted in Butterfield’s Buffer (3 M US; Minneapolis, MN) at undiluted, 1:10, 1:100, 1:1,000, 1:10,000 and 1:100,000 dilutions before 1 mL aliquots of each dilution were plated onto room temperature aerobic and *E. coli* Petrifilm™. The film was placed onto the product agar and the sample distributed using a plate spreader. The agar was allowed to solidify for 1 min and then incubated at 32 ± 2 °C for 48 ± 2 h. As a control, buffer alone was inoculated as described above to confirm diluent sterility. For total aerobic count, red colonies were enumerated from the aerobic Petrifilm™. For the total coliform count, all red and blue colonies with gas were enumerated from the *E. coli* plate. For the *E. coli* count, only blue colonies with gas were enumerated from the *E. coli* Petrifilm™ and reported as colony forming units per mL. For full methodological description of measurement of colostrum neutralizing titers see Serum Analysis section below.

### Serum analysis

Serum samples were examined for total protein (TP, day 3 only), IgG concentration, serum neutralizing titers (SNTs) against IBRV, BVDV Types 1 and 2, and SNTs against five common serovars of *Leptospira spp.* (*canicola*, *grippotyphosa*, *hardjo*, *icterohaemorrhagiae* and *pomona*). Measurement of serum TP was by refractometry (Reichert®, Reichert Analytical Instruments; Seefeld, Germany).

The concentration of IgG in serum was determined spectrophotometrically by turbidimetric immunoassay for bovine serum/plasma analogous to the technique described above for colostrum. Apparent efficiency of IgG absorption (AEA) was calculated using the following equation [57, 58]:

$$AEA= \left\{\frac{\mathrm{Serum\ IgG\ concentration }\ (\mathrm{g}/\mathrm{L})\ \mathrm{ x\ BW }\ (\mathrm{kg})\ \mathrm{ x }\ 0.09}{\mathrm{Colostral\ IgG\ concentration }\ (\mathrm{g}/\mathrm{L})\mathrm{ x }4}\right\}\mathrm{x }100$$

Where 0.09 = estimated plasma volume as % of body weight and 4 = the volume of colostrum administered in L; colostral IgG concentration x volume fed = IgG intake in g.

Neutralizing titers were determined by virus neutralization (VN) assays to detect serum and colostral antibodies directed against IBRV, and BVDV Types 1 and 2. After heat inactivation at 56 °C, sera were diluted in duplicate across 96-well flat-bottomed microtiter plates (Corning Life Sciences DL; Corning, NY), resulting in dilutions ranging from 1:2 up to 1:4096. Diluted samples were incubated with an equal volume of virus at 200 × TCID50 (tissue culture infectious dose 50) per 50 µl for 60 min. Viruses used included IBRV and cytopathic BVDV Types 1 and 2. A control plate was included in each run and contained known positive and negative sera, viral back-titer, and cell control. After initial incubation, a suspension of Madin-Darby bovine kidney (MDBK) cells was added to all wells. A further wash step was added for colostral samples after 1 h. All plates were then incubated at 37 °C and 5.5% CO_2_ for 3 days. Plates were read on an inverted microscope. Endpoint titer was determined as the dilution at which neutralization of virus ended, as detected by viral cytopathic effect (CPE).

Serum neutralizing titers against leptospiral serovars were determined using *Leptospira interrogans* serovar *canicola* (strain Hond Utrecht IV), serovar *grippotyphosa* (strain Andaman), serovar *hardjo* (strain Hardjoprajitno), serovar *copenhageni* (serogroup Icterohaemorrhagiae; strain M20), and serovar *pomona* (strain Akiyami A) which were cultured and subcultured weekly in 10 mL of polysorbate 80-bovine albumin liquid broth (P80-BA, National Veterinary Service Laboratories; NVSL, Ames, IA) incubated at 28 ± 2 °C. Weekly, approximately 100 µL was cultured on a blood agar plate (Becton Dickinson; Franklin Lakes, NJ) and grown at 35 ± 2 °C for 48 h to check for contamination. Antigen for the *Leptospira* microagglutination (LeptoMAT) test were prepared as follows. *Leptospira* cultures were centrifuged at 600 × g for 10–15 min, supernatant was transferred to an 18 × 150 mm glass tube and analyzed for transmittance using a spectrophotometer (Spectronic 20D, Milton Roy; Rochester, NY). Antigen density must range between 60 – 70% transmittance at a single-beam wavelength of 400 nm and can be adjusted using sterile P80-BA. Homologous reference positive control serum (NVSL) for each antigen/serovar was used at a 1:50 dilution in 0.01 M phosphate-buffered saline (PBS, pH 7.2 – 7.6) to confirm the specificity of the antigen. Serum to be tested was diluted initially 1:50 in PBS (50 µL) and serially diluted to 1:102,400 as needed in a flat-bottom 96-well microtiter plate. 50 µL of each antigen was added to each well of diluted sera or homologous reference positive control sera creating a 1:100 dilution in the first sample well as well as for the positive control serum. Micro-titer plates were incubated 1 h and 20 min to 2 h at room temperature. Each micro-titer plate was observed under the dark-field microscope (DM1000, Leica; Wetzlar, Germany) using 20 × magnification with an indirect light source and the agglutination between antibodies and the free-swimming *Leptospira* was observed. Agglutination was semi-quantitatively assessed using the following metrics: 1) negative is no agglutination, 2) trace is 1–24% agglutination and up to 75% free-swimming *Leptospira*, 3) + 1 agglutination is 25–49% agglutination, 4) + 2 agglutination is 50–74% agglutination, 5) + 3 agglutination is 75–99% agglutination and 6) + 4 agglutination is 100% agglutination with no free-swimming *Leptospira* observed. Positive samples (2 + , 3 + , and 4 + agglutination) are reported according to their end-point titer. Reference positive control serum must obtain agglutination (+ 2, + 3 or + 4 agglutination) with the homologous serovar in at least the 1:3,200 dilution for the test to be valid. The negative control containing just *Leptospira* and PBS must be negative for each serovar.

### Statistical analysis

Prior to the start of the study a power calculation was performed focusing on the ability to detect differences in titers between groups. The criteria for the power calculation assumed only three time-sampling points and were set to detect a minimum titer difference of 1:291 between the MC and AC groups with alpha = 0.05 and power = 100. Data generated by the study were analyzed using STATA 17.0 (Stata Corp., College Station, TX). Descriptive statistics (mean, standard deviation, median, range, and interquartile range [IQR 25%-75%]) were calculated for all outcome variables. Normality was assessed by a Shapiro Wilk test. A t-test was used to evaluate associations between feeding MC or AC and outcome variables where data were normally distributed. Non-normal data were checked with a series of transformations but could not be normalized, hence the Wilcoxon rank sum or Kruskal Wallis tests, which make no assumptions about data distributions, were used to examine untransformed continuous or interval outcome variables, respectively. For serum IgG concentrations, regression analysis was used to compare the effect of MC and AC over the entire time course of days 3–180. Colostrum IgG concentrations measured by colostrometer and Brix refractometer were compared to those obtained by TIA using pairwise Pearson correlation coefficients, as were serum total protein and serum IgG concentration measured on day 3 by refractometry and TIA, respectively. Using parameters from regression analysis (i.e., coefficient and intercept) cutoffs were determined for the colostrometer and Brix refractometer readings corresponding to 50 g/L of IgG as measured by TIA. The same technique was used to determine the serum immunoglobulin concentration cutoffs measured by TIA that were equivalent to serum total protein levels of 5 and 5.5 mg/dL. Because a large number of SNT outcomes were below the detection limit of the various neutralization assays, where appropriate tobit regression was used. The latter estimates relationships between variables when upper and lower censoring occurs such as with tests that have both lower and upper detection limitations. Inclusion of calf as a fixed effect and robust variance estimation were used to control for clustering in all regressions. Statistical significance was inferred when *P* < 0.05.

## Data Availability

Open Science Framework (OSF) repository, accession number https://doi.org/10.17605/OSF.IO/3XNZQ at the publicly accessible link https://osf.io/3xnzq/

## Abbreviations

IgG: Immunoglobulin G
MC: Untreated maternal colostrum
AC: Formic acid acidified maternal colostrum
IBRV: Infectious bovine rhinotracheitis virus
BVDV: Bovine viral diarrhea virus
cfu: Colony forming units
SD: Standard deviation
AEA: Apparent efficiency of IgG absorption
SNT: Serum neutralizing titer(s)
TIA: Turbidometric immunoassay
RID: Radial immunodiffusion
FDA: United States Food and Drug Administration
NVSL: National Veterinary Service Laboratories
PBS: Phosphate-buffered saline
IQR: Interquartile range 25^th^-75^th^ percentile

## Footnotes

1 Value Diagnostics, DVM Rapid Test™ II—Multi-Test Analyzer Bovine Test Kits: IgG Colostrum and. IgG Serum / Plasma; available from MAI™ Animal Health, Elmwood WI, USA.
